# Profils épidemiologiques, cliniques et hématologiques de la drépanocytose homozygote SS en phase inter critique chez l’enfant à Ziguinchor, Sénégal

**DOI:** 10.11604/pamj.2017.28.208.14006

**Published:** 2017-11-07

**Authors:** Lamine Thiam, Assane Dramé, Isabelle Zokébé Coly, François Niokhor Diouf, Ndiogou Seck, Djibril Boiro, Aliou Abdoulaye Ndongo, Idrissa Basse, Babacar Niang, Indou Deme/Ly, Assane Sylla, Ibrahima Diagne, Ousmane Ndiaye

**Affiliations:** 1 Université Assane Seck de Ziguinchor, Hôpital de la Paix de Ziguinchor; 2 Université Assane Seck de Ziguinchor, Centre Hospitalier Régional de Ziguinchor; 3 Université Gaston Berger de Saint Louis, Hôpital Régional de Saint Louis; 4 Université Cheikh Anta Diop de Dakar, Hôpital d’Enfants Albert Royer de Dakar; 5 Université de Thiés, Centre Hospitalier National d’Enfants de Diamniadio; 6 Université Cheikh Anta Diop de Dakar, Hopital Aristide Ledantec

**Keywords:** Drépanocytose SS, phase inter critique, enfant, Sickle cell diasease SS, intercritical period, child

## Abstract

La drépanocytose pose un problème de santé publique au Sénégal. Elle concerne principalement les enfants et les adolescents. L'objectif de notre travail était de déterminer les profils épidémiologiques, cliniques et hématologiques de la drépanocytose homozygote SS dans une cohorte d'enfants suivis à l'hôpital de la paix de Ziguinchor. Il s'agit d'une étude rétrospective portant sur des dossiers d'enfants drépanocytaires. Etaient inclus, les drépanocytaires SS âgés entre 2mois et 21ans, reçus en phase inter critique durant la période d'étude allant du 1^er^ Janvier 2015 au 31 Août 2017. N'étaient pas inclus dans l'étude les hétérozygotes composites (SC, S béta thalassémie). Nous avons colligé 46 dossiers de drépanocytaires SS (20 filles et 26 garçons). L'âge moyen des enfants était de 8,0ans [11mois-21ans]. Environ 1/3 des enfants (39,1%) avaient un âge inférieur ou égal à 5 ans. Il y avait une diversité ethnique avec une prédominance de diola (30,2%) suivi de mandingue (27,9%) et de poular (25,6%). L'âge moyen des enfants à la 1ére crise était de 35,5mois [7-192 mois]. Plus de 1/3 des enfants (41,3%) avaient fait une 1ére crise avant le deuxième anniversaire. Le type de la 1ére crise, qu'avait présenté l'enfant, était dominé par la crise vaso-occlusive (32,6%) suivi du syndrome pied-main (30,4%). Les signes cliniques en phase inter critique étaient la pâleur 95,6%); l'ictère (36,9%) et la splénomégalie (21,7%). A l'hémogramme, le nombre de globules blancs moyen était de 12465 leucocytes/mm^3^ [5340-26900]. L'hyperleucocytose, supérieure à 10 000 leucocytes/mm^3^ était retrouvée chez 34 malades (73,9%). La totalité des malades présentait une anémie avec une moyenne 08,6 g/dl [05,7-11,8]. Le taux d'hémoglobine S variait entre 54,6 et 98,4%. Le diagnostic et la prise en charge médicale de la drépanocytose SS sont tardifs à Ziguinchor. Le dépistage néonatal pourrait favoriser une prise en charge précoce dans la région.

## Abstract (en)

Sickle cell disease poses a public health problem in Senegal. It mainly affects children and adolescents. This study aimed to determine the epidemiological, clinical and hematological profiles of homozygous (SS) sickle cell disease in a cohort of children followed-up at the Peace Hospital in Ziguinchor. We conducted a retrospective study of the medical records from children with sickle cell disease. All patients aged between 2 months and 21 years with sickle cell disease SS during the intercritical period, hospitalized during the study period from 1^st^ January 2015 to 31 August 2017 were included in our study. Compound heterozygous patients (SC, S Beta Thalassemia) were not included. We collected 46 medical records of patients with sickle cell disease SS (20 girls and 26 boys). The average age of children was 8,0 years [11 months-21 years]. Approximately 1/3 of children (39.1%) had an age less than or equal to 5 years. There was an ethnic diversity showing a predominance of the Diola (30.2%) followed by the Mandinga (27.9%) and the Poular (25.6%). The average age of children with first crisis was 35,5 months [7-192 months]. More than 1/3 of children (41.3%) had had first crisis before their second anniversary. In the child, first crisis was dominated by vaso-occlusive crisis (32.6%) followed by hand-foot syndrome (30.4%). Clinical signs during the intercritical period were pallor 95.6%), jaundice (36.9%) and splenomegaly (21.7%). Mean white blood cell count was 12465 leucocytes/mm^3^ [5340-26900]. Hyperleukocytosis greater than 10 000 leucocytes/mm^3^was found in 34 patients (73.9%). All patients had anemia with an average hemoglobin of 08,6 g/dl [05,7-11,8]. Hemoglobin S rate ranged between 54.6 and 98.4%. Diagnosis and medical management of sickle cell disease SS are delayed in Ziguinchor. Neonatal screening may lead to improve early management of patients in the region.

## Introduction

La drépanocytose est une maladie héréditaire de l'hémoglobine. Selon l'OMS, près de 5% de la population mondiale, sont porteurs d'un gène responsable d'une anomalie de l'hémoglobine [1]. La majorité des personnes atteintes de cette maladie, vive en Afrique noir avec des prévalences qui varient entre 10 et 40% [2]. Elle pose un problème majeur de santé publique dans ces pays en développement. Au Sénégal, 1 personne sur 10, sans distinction d'ethnie, d'origine géographique ou de classe sociale, porte le gène de la drépanocytose. La majorité ne l'a hérité que d'un des parents et ne présente aucun signe. Ce sont les porteurs du trait drépanocytaire (AS). Cependant, de leur union naissent les enfants porteurs de la drépanocytose homozygote (SS) avec 25% des risques à chaque grossesse. Ainsi, environ 1700 enfants naissent, chaque année, avec la drépanocytaire au Sénégal. La forme homozygote SS se manifeste par une anémie, une susceptibilité aux infections et par des crises douloureuses osseuses et/ou abdominales. En l'absence de prise en charge approprié de cette forme SS, 50% des enfants décèdent avant l'âge de 5 ans. C'est pourquoi le pays s'est engagé dans la lutte contre la drépanocytose, et depuis plus de 30ans la prise en charge médicale s'organise de plus en plus dans les structures hospitalières à travers la création de centres de suivis ambulatoires. L'objectif de notre travail était de déterminer les profils épidémiologiques, cliniques et hématologiques de la drépanocytose SS en phase inter critique dans une cohorte d'enfants suivis à l'hôpital de la paix de Ziguinchor.

## Méthodes

**Site de l'étude:** Notre étude avait eu pour cadre le service de pédiatrie de l'hôpital de la paix de Ziguinchor. L'hôpital est une structure de référence dans la région de Ziguinchor et dans la sous-région. Il comporte un service de pédiatrie d'une capacité de 30 lits. Le personnel médical et paramédicale de ce service est constitué par: un pédiatre assistant universitaire, deux autres pédiatres praticiens hospitaliers, cinq infirmiers d'état et sept assistants infirmiers. L'hôpital de la paix de Ziguinchor est un hôpital universitaire de niveau 3 qui comporte entre autre un laboratoire d'analyse bactériologique, biochimique et hématologique.

**Type d'étude:** Il s'agissait d'une étude rétrospective descriptive et analytique portant sur des dossiers d'enfants drépanocytaires SS suivis dans le service de pédiatrie de l'hôpital de la paix de Ziguinchor, durant la période allant du 1^er^ janvier 2015 au 31 Août 2017.

**Population d'étude:** Elle concernait l'ensemble des enfants drépanocytaires homozygote SS suivi régulièrement dans le service de pédiatrie de l'hôpital de la paix de Ziguinchor.

**Critères d'inclusions:** Étaient inclus dans l'étude, les enfants drépanocytaires homozygotes SS âgés entre 2 mois et 21 ans, reçus en phase inter critique durant la période d'étude.

**Critères de non inclusions:** N'étaient pas inclus dans l'étude les hétérozygotes composites (SC, S béta thalassémie); les enfants âgés de moins de 2 mois.

**Définition de cas:** La phase inter critique était définit par l'absence de fièvre, de douleur osseuse ou abdominale et d'hémolyse aigue.

**Recueil des données:** Les paramètres étudiés, étaient les données sociodémographiques et épidémiologiques (âge, sexe, niveau socio-économique, origine ethnique, consanguinité parentale); les antécédents médicaux (première crise, circonstance du diagnostic, nombre d'hospitalisation, nombre de transfusion); les données cliniques (appréciation de l'ictère; de la pâleur et la classification de la splénomégalie selon Hackett); les données biologiques (hémogramme, taux de réticulocytes, taux d'hémoglobine S, A2 et F). Pour chaque patient, les variables épidémiologiques, cliniques et hémaologiques ont été recueillies sur une fiche d'enquête puis analysées grâce au logiciel Epi-Info.

## Résultats

Durant la période d'étude, nous avons colligé 46 dossiers d'enfants drépanocytaires homozygotes SS, dont 20 filles et 26 garçons. L'âge moyen des enfants pendant la période d'étude était de 8,0ans [11mois-21ans]. Environ 1/3 des enfants (N=18 soit 39,1%) avaient un âge inférieur ou égal à 5 ans et 07 (soit 15,2%) enfants étaient âgés de moins de 2 ans.

**Les données socio démographiques et épidémiologiques:** Elles sont représentées dans le **Tableau 1**.

**Tableau 1:** Données sociodémographiques et épidémiologiques

	Variable	Effectif	Pourcentage
**Age**			
	≤ 5 ans	18	39,1
	6-10 ans	11	23,9
	11-15 ans	14	30,4
	> 15 ans	03	06,5
**Sexe**			
	Masculin	26	56,5
	Féminin	20	43,5
**Niveau socio-économique**			
	Bas	32	69,6
	Moyen	10	21,7
	Elevé	04	08,7
**Ethnie**			
	Diola	13	30,2
	Mandingue	12	27,9
	Poular	11	25,6
	Wolof	3	07,0
	Sérer	2	04,7
	Autres	2	04,6
**Origine géographique**			
	Rurale	08	17,4
	Sub urbaine	06	13,0
	Urbaine	32	69,6
**Consanguinité**			
	Oui	18	41,9
	Non	25	58,1

**Les données cliniques:** L'âge moyen des enfants à la première crise était de 35,5mois [7-192 mois]. Plus de 1/3 (N=19, soit 41,3%) des enfants avaient fait une première crise durant les deux premières années de vie. Le type de la première crise, qu'avait présenté l'enfant, est représenté dans le Tableau 2. La moyenne d'hospitalisation était de 1 avec des extrêmes allant de 0 à 4. Trente-six malades (soit 78,3%) avaient déjà étaient hospitalisés au moins une fois. La moitié des malades avaient été déjà transfusé avec une moyenne de transfusion de 0,5 et des extrêmes allant de 0 à 4. Les signes cliniques en phase stationnaire sont représentés dans le Tableau 3.

**Tableau 2:** Répartition des enfants selon le type de la première crise

Type de la première crise	Effectif	Pourcentage
**Crise vaso-occlusive (CVO)**	15	32,6
**Syndrome pied-main**	14	30,4
**Anémie sévère**	07	15,2
**Infection**	04	08,7
**Séquestration splénique**	03	06,5
**Priapisme**	02	04,3
**Syndrome thoracique aigue (STA)**	01	02,2
**Total**	46	100

**Tableau 3:** Répartition des enfants selon les signes cliniques

Signes cliniques	Effectif	Pourcentage
**Pâleur**	44	95,6
**Ictère**	17	36,9
**Splénomégalie**	10	21,7

**Les données biologiques:** Les circonstances du diagnostic biologique de la drépanocytose étaient une hospitalisation dans 67,4% des cas (N=31); des symptômes cliniques dans 19,6% des cas (N=09) et un dépistage familial dans 13,0% des cas (N=06). L'âge moyen au diagnostic biologique était de 60 mois [1-252mois]. Vingt-quatre malades ont été diagnostiqués à un âge inférieur ou égal à 5 ans et 22 malades à un âge supérieur à 5 ans. Les données biologiques sont représentées dans le Tableau 4. L'hyperleucocytose, supérieure à 10 000 leucocytes/mm^3^ était retrouvée chez 34 malades (soit 73,9%). La totalité des malades présentait une anémie, qui était normocytaire dans 50% des cas (N=23) et microcytaire dans 50% des cas (N=23). L'anémie était normo chrome dans 69,6% des cas (N=32) et hypochrome dans 30,4% des cas (N=14). Elle était régénérative dans 71,7% des cas (N=33) et arégénérative dans 28,3% des cas (N=13).

**Tableau 4:** Données biologiques

Données biologiques	Valeur moyenne	Valeurs extrêmes
**Leucocytes (mm^3^)**	12465	5340 - 26900
**Taux d’hémoglobine (g/dl)**	08,6	05,7 - 11,8
**VGM (fl)**	84,8	48,3 - 92,3
**TCMH (pg)**	29,1	14,1 - 36,9
**CCMH (g/dl)**	38,1	29,1 - 43,4
**Taux de réticulocytes (mm^3^)**	121 200	9240 - 331 500
**Hémoglobine S (%)**	86,8	54,6 - 98,4
**Hémoglobine A2 (%)**	02,2	01,1 - 45,3
**Hémoglobine F (%)**	04,0	00,0 - 37,6

## Discussion

**Les données socio démographiques et épidémiologiques:** L'âge moyen de nos patients pendant la période d'étude était de 8,0 ans. Environ 1/3 des malades (N=18) étaient âgés de moins de 5 ans et seulement 07 enfants étaient âgés de moins de 2 ans. Au Sénégal, le diagnostic de la drépanocytose est rarement posé avant l'âge de 2 ans et le dépistage néonatal n'est pas systématique [3]. La découverte précoce de la drépanocytose dans notre milieu de travail est fonction de la précocité des signes d'appel expliquant ainsi la faible fréquence observée des drépanocytaires âgés de moins de 2 ans dans notre série (N=07, soit 15,2%). Des techniques fiables de dépistage néonatal sont disponibles depuis plus de 40 ans et se perfectionnent davantage. La systématisation du dépistage néonatale dans la région de Ziguinchor permettrait de dépister précocement la majorité des hémoglobinopathies notamment la drépanocytose. Le sexe masculin représentait un peu plus de la moitié des cas (56,5%) dans notre étude. Cette prédominance masculine est aussi retrouvée dans l'étude de Boiro, qui dans sa série avait trouvé un sexe ratio de 1,42 [4]. Par contre, d'autres auteurs dont Nacoulma et Samira rapportent une légère prédominance féminine [5,6]. Enfin, d'autres auteurs ne constatent aucune prédominance entre les deux sexes; c'est le cas de Thuilliez [7] et de Dreux [8]. Ces différences seraient en rapport avec les données démographiques de chaque pays car la transmission de la drépanocytose n'est pas liée au sexe [9,10]. Les résultats de notre étude montrent une diversité ethnique au niveau de la région de Ziguinchor avec une prédominance de diola (30,2%) suivi de mandingue (27,9%) et de poular (25,6%). Plus de deux tiers des malades sont de famille ayant un bas niveau socioéconomique. Ces résultats corroborent avec ceux du service régional de la prévention et de la statistique de la région de Ziguinchor [11].

**Le tableau clinique:** Un tiers (30,4%) des drépanocytaires de notre série ont présenté un syndrome pied-main comme première crise drépanocytaire et 41,3% ont présentaient leur première crise avant d'atteindre leur deuxième anniversaire. Ces résultats sont moins importants que ceux de Shongo qui retrouvait dans sa série 56,1% de syndrome pied-main comme première crise drépanocytaire et 82,9% d'enfants ayant présenté leur première crise avant d'atteindre leur premier anniversaire [12]. Tshilolo retrouve les signes d'appel dans 80% avant l'âge de 12 mois; dans ses formes à révélation précoce, il retrouve le syndrome pied-main et/ou l'anémie [3]. La différence pourrait s'expliquer par les haplo types de la drépanocytose. La moitié de nos malades ont été transfusé au moins une fois. Ces résultats sont comparables à ceux de la littérature [5, 13-15]. Dans la série de Shongo, près de 80% des patients ont reçu au moins une transfusion [12]. De Montalembert rapporte que plus de 60% des patients homozygotes SS sont transfusés au moins une fois avant leur 18ème anniversaire [16]. Quatre-vingt-quinze virgule six pour cent (95,6%) des malades présentaient une pâleur, ce qui témoigne de l'anémie chronique chez les enfants drépanocytaires. Da Silva retrouvait 51,5% de pâleur cutanéo-muqueuse dans une série de 33 enfants drépanocytaires au Maroc [17]. L'ictère était observé dans environ 1/3 de cas (36,9%). Shongo retrouve, dans sa série, l'ictère dans 63,4% des cas [12]. L'ictère est dû à l'hémolyse importante que l'on rencontre dans la drépanocytose. Il faut signaler que l'ictère apparaît consécutivement à l'anémie et classiquement après l'âge de 6 mois, âge auquel l'hémoglobine fœtale commence à être remplacée par l'hémoglobine S qui devient alors prédominante [18]. La splénomégalie était présente chez 10 patients parmi lesquels 4 étaient âgés de moins de 5 ans et 6 étaient âgés de plus de 5 ans. Ces résultats sont contradictoires à l'hypothèse de la régression splénique après l'âge de 5 ans chez les enfants drépanocytaires [19]. La persistance de la splénomégalie après l'âge de 5 ans retrouvé dans notre étude a déjà été signalée dans d'autres études africaines [20,21]. En effet Adekile soutient la thèse d'une possible réaction avec le paludisme, ce qui pourrait être le cas dans notre série puisque la région naturelle de la Casamance est caractérisée par une forte prévalence du paludisme [20].

**Les données biologiques:** Dans notre étude, l'hémogramme était pratiqué de façon systématique pour les malades. L'anémie était retrouvée chez tous les enfants. Le taux d'hémoglobine variait entre 05,7 à 11,8 g/dl, rejoignant les études faites par Samira [5]; Bouzaid [14] et Harack [22]. L'anémie était normo chrome normocytaire dans plus de la moitié des cas (69,6%) et régénérative dans 71,7% des cas. Elle s'explique par une hémolyse chronique chez l'enfant drépanocytaire. Plus de la moitié de nos drépanocytaires (N=24) avaient une hyperleucocytose supérieure à 10 000 leucocytes/mm^3^ avec une moyenne de 12 465 éléments/mm^3^. En effet l'hyperleucocytose est physiologique dans la drépanocytose et s'expliquerait par l'hyper activité de la moelle et les phénomènes inflammatoires [23,24]. Des résultats similaires sont retrouvés dans la littérature [14, 15, 25].

## Conclusion

La drépanocytose SS pose un problème de santé publique au Sénégal. Le diagnostic et la prise en charge médicale sont tardifs à Ziguinchor. Le dépistage néonatal pourrait améliorer le diagnostic et favoriser une prise en charge précoce dans la région.

### Etat des connaissances actuelle sur le sujet

- La drépanocytose pose un problème de santé publique en Afrique noir en général e au Sénégal en particulier;
- Le mnistère de la santé du Sénégal s'est engagé dans la lue contre la drépanocytose;
- La création de centres de recherche et de suivi ambulatoire de la drépanocytose à Sain Louis (nord du Sénégal) e à Dakar (centre ouest du Sénégal).

### Contribution de notre étude à la connaissance

- Compare les données épidémiologiques, cliniques et biologiques d'une cohorte d'enfants drépanocytaires de Ziguinchor avec les données de la littérature;
- Renseigne sur les données épidémiologiques dans la région sud du Sénégal;
- Sert d'étude pilote pour la création d'un centre de suivi ambulatoire de la drépanocytose au Sud du Sénégal.

## Conflits d’intérêts

Les auteurs ne déclarent aucun conflit d'intérêts.
